# Cellular organization and histogenesis of adenosquamous carcinoma of the pancreas: evidence supporting the squamous metaplasia concept

**DOI:** 10.1007/s00418-020-01864-y

**Published:** 2020-03-13

**Authors:** Werner Boecker, Katharina Tiemann, Joerg Boecker, Marieta Toma, Michael H. Muders, Thomas Löning, Igor Buchwalow, Karl J. Oldhafer, Ulf Neumann, Bernd Feyerabend, Andre Fehr, Göran Stenman

**Affiliations:** 1 Gerhard-Domagk Institute of Pathology, University of Münster, Münster, Germany; 2 Department of Pathology/Hematopathology, Institute for Hematopathology, Fangdieckstr. 75, 22547 Hamburg, Germany; 3 Department of Surgery and Transplantation, University Hospital RWTH Aachen, Aachen, Germany; 4 Institute of Pathology, University of Bonn, Bonn, Germany; 5 Gerhard-Seifert Reference Center for Oral-, Gyneco-, and Breast Pathology, Hamburg, Germany; 6 Department of Surgery, Asklepius Clinic Barmbek, Hamburg, Germany; 7 Department of Pathology, Asklepius Clinic Barmbek, Hamburg, Germany; 8 Sahlgrenska Cancer Center, Department of Pathology, University of Gothenburg, SE- 405 30 Gothenburg, Sweden

**Keywords:** Adenosquamous carcinoma, Pancreas, Keratins, p63, p40, MUC1, MUC5AC, Ki67, Transdifferentiation

## Abstract

Adenosquamous carcinoma of the pancreas (ASCAP) is characterized by conventional pancreatic ductal adenocarcinoma (PDAC) and squamous carcinoma components with at least 30% of the tumour showing squamous differentiation. To get further insight into the histogenesis of these lesions, we analysed the cellular organization of ASCAP compared to PDACs. Using Immunohistochemistry and triple immunofluorescence labelling studies for keratins, p63, p40, MUC1, MUC2, MUC5AC, Ki67, and EGFR we demonstrate that many ASCAPs contain a transitional zone between the K8/18-positive adenocarcinomatous component and the p63+ /p40+ /K5/K14+ squamous component initiated by the expression of p63 in K8/18+ adenocarcinomatous cells and the appearance of basally located p63+ K5/14+ cells. p63+ K5/14+ cells give rise to fully developed squamous differentiation. Notably, 25% of conventional PDACs without histologically recognizable squamous component contain foci of p63+ p40+ and K5/14+ cells similar to the transitional zone. Our data provide evidence that the squamous carcinoma components of ASCAPs originate from pre-existing PDAC via transdifferentiation of keratin K8/18-positive glandular cells to p63-, p40-, and keratin K5/14-positive squamous carcinoma cells supporting the squamous metaplasia hypothesis. Thus our findings provide new evidence about the cellular process behind squamous differentiation in ASCAPs.

**Electronic supplementary material:**

The online version of this article (10.1007/s00418-020-01864-y) contains supplementary material, which is available to authorized users.

## Introduction

Adenosquamous carcinoma of the pancreas (ASCAP) is a rare histological subtype of pancreatic ductal adenocarcinoma (PDAC) (Boyd et al. 2012; Gill et al. 2019; Imaoka et al. 2014; Katz et al. 2011; Murakami et al. 2003; Okabayashi and Hanazaki 2008; Simone et al. 2013; Voong et al. 2010). According to the WHO-classification of pancreatic carcinomas, ASCAP is characterized by variable proportions of glandular and squamous carcinoma component and the squamous component should account for at least 30% of the tumour to qualify as ASCAP (Gill et al. 2019).

Several hypotheses have been proposed regarding the histogenesis of ASCAP, including the differentiation-, the squamous metaplasia-, and the collision theory (Borazanci et al. 2015; Kardon et al. 2001; Madura et al. 1999; Motojima et al. 1992). According to the differentiation theory, the squamous and adenocarcinoma components of ASCAP develop from the same progenitor cancer cells. This theory is supported by recent molecular findings demonstrating similar genomic alterations in the two components of ASCAP (Fang et al. 2017; Marcus et al. 2017). A similar model was recently proposed for low grade adenosquamous carcinoma/syringoma of the breast, tumours which also contain squamous and glandular elements (Boecker et al. 2015, 2017). The squamous metaplasia theory suggests that the malignant squamous component of ASCAP originate from pre-existing PDAC (Kardon et al. 2001; Yamaguchi and Enjoji 1991). Finally, the collision theory, suggesting that the glandular and squamous components arise independently from each other and subsequently merge to one tumour, and the proposal that squamous metaplasia of the pancreatic ductal epithelium, occurring in the setting of chronic pancreatitis, contributes to the development of ASCAP (Simone et al. 2013), are nowadays abandoned by most authors (Kardon et al. 2001).

We hypothesize that the cellular organization of these tumours gives us further insight into the histogenesis of these lesions. We, therefore, analysed the cellular organization of 25 ASCAPs compared to 20 PDACs. We used immunohistochemistry and triple immunofluorescence stainings for p63, p40, different keratins, MUC1, MUC2, MUC5AC, the proliferation marker Ki67, and EGFR. Notably, we detected transdifferentiation of pre-existing glandular elements to squamous elements and subscribe to the theory that adenosquamous carcinomas in the pancreas occur as a result of malignant squamous metaplastic change of an adenocarcinoma.

## Material and methods

### Case selection

Among 562 patients with PDAC (Table 1), who had undergone pancreas resection with curative intent from 2007–2018, twenty-five cases (4.4%) were diagnosed as ASCAP with the squamous component accounting for at least 30% of the tumour (Gill et al. 2017). The samples were redundant clinical specimens that had been de-identified and unlinked from patient information. Formalin-fixed paraffin-embedded (FFPE) tissue blocks were retrieved from these cases and from 20 cases of PDAC from the files of the Departments of Pathology of the University of Aachen and of the Asklepius Clinic Hamburg Barmbek. As most of these tumours were classified according to the 7th edition of UICC TNM classification (Sobin et al. 2009) we used this edition for all cases. The study was approved by the Committee for Research Ethics (review board) of the University of Aachen and by the Ethic Board of the Institute of Hematopathology Hamburg.

**Table 1:** Demographic data, morphological findings, and outcome of 25 patients with ASCAP

Features	
Gender (m/f)	10/ 15
Age (median)	71 (55–81 years)
Site	
Head	18 (72%)
Tail	7 (28%)
Size (median)	3.92 (range 1.7–7.0 cm)
Histologic grade	
Grade 1	0
Grade 2	7 (28%)
Grade 3	15 (60%)
Grade 4	3 (12%)
Mitosis per 10HPF	Mean 5.72
Squamous component (mean %)	74
TNM	
pT1	1 (4%)
pT2	–
pT3	21 (84%)
pT4	3 (12%)
pN1	16 (64%)
M1	4 (16%)
L1	19 (76%)
V1	11 (44%)
No of nodes analysed/case	17.95 (SD 8, range 31)
Pn1	22 (88%)
R1	12 (48%)
PanIN	13 (52%)
Overall survival (median)	8.2 months

### Histopathology and immunohistochemistry

Histological grading of the ductal component was performed according to the current WHO-classification of pancreatic tumours (Gill et al. 2019). Carcinomas containing a large amount of anaplatic/sarcomatoid differentiations were classified as grade 4. FFPE tissue sections (4 μm thick) were pre-treated and stained as described elsewhere (Buchwalow et al. 2011). Immunostains and triple immunofluorescence stainings were performed using antibodies raised against different keratins, p63, p40, CEA, EGFR, MUC1, MUC2, MUC5AC, p53, and Ki67. The sources and dilutions of all antibodies are shown in Table 2. The four different antibodies against the high-molecular weight keratins K5 and K14 (Table 2) yielded identical results, so that the term “K5/14” is used throughout the text. All markers were scored separately and quantified as percentages of the total number of tumour cells counted in 25 cases of ASCAP and 20 cases of PDAC.

**Table 2:** Primary antibodies used in this study

Antibody	Clone	Source	Dilution
p63	4A4	Biocare medical	1:50
K5	ER16014	MEDAC	1:100
K5/6	D5/16 B4	Dako	1:50
K14	LL002	AbCam	1:50
K5/14	EP160Y/LL002	Cell marque	Ready to use
K7	OV-TL12/30	Dako	1:50
K18-FITC	CY-90	Sigma	1:50
K8/18	5D3	Zytomed	1:50
EMA	E 29	Ventana	Ready to use
MUC1	NCL-MUC1 Ma695	Novocastra	1:50
MUC2	NCL-MUC2 CcP58	Novocastra	1:50
MUC5AC	MRQ-19	Cell marque	Ready to use
EGFR	3C6	Ventana	Ready to use
Ki67	SP6	Thermo fisher	1:100

## Results

### Clinicopathological features

The clinico-pathological characteristics of the patients with ASCAP, including 15 females and 10 males, are shown in Table 1. The median age of the patients was 71 years (range 55–81) and the median size of the tumors was 3.92 cm (range 1.7–7.0 cm). Eighteen tumors were located in the head and seven in the tail of the pancreas. The tumors had a mean squamous component of about 70% and were graded 2 (28%), 3 (60%), or 4 (12%). The median overall survival of the patients was 8.2 months.

### Transitional zones occur between the adenocarcinomatous and squamous carcinoma components

**Fig. 1:**
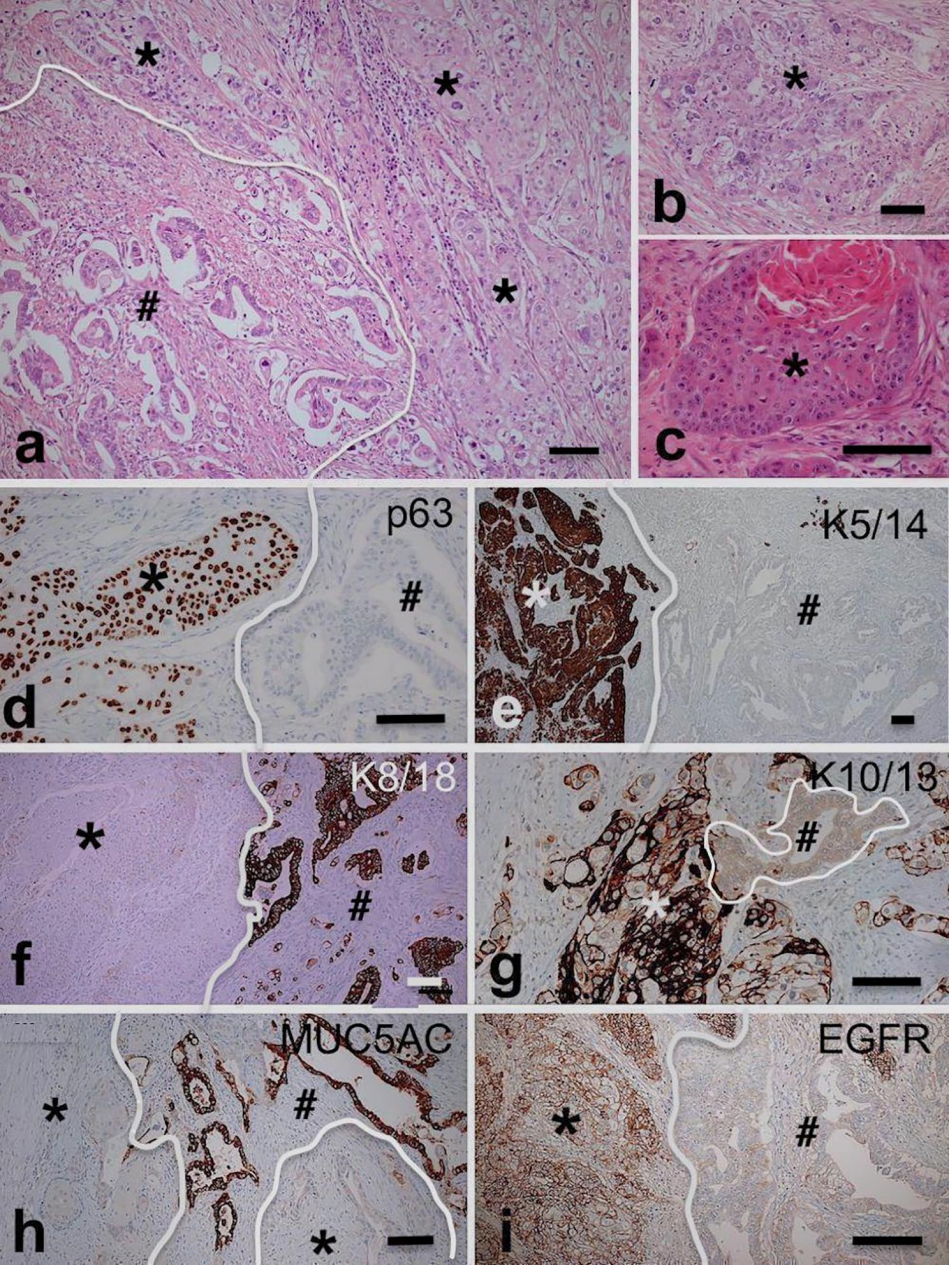
Histological and immunohistochemical features of adenosquamous carcinoma of the pancreas; all pictures are derived from the same tumour **a** Classical patchwork pattern with abrupt transition between adenocarcinomatous (hashtag) and squamous elements (asterisks); **b**–**c** higher magnification of squamous elements of the same tumour with nonkeratinizing (**b**) and keratinizing features (**c**); **d**–**i** immunostainings showing positivity for K8/18 and Muc5AC in the glandular component (hashtags in **f** and **h**) and diffuse robust staining for p63, K5/14, and EGFR in the squamous component (asterisks in **d**, **e**, **g**, and **i**). Scale bar 100 µm

**Fig. 2:**
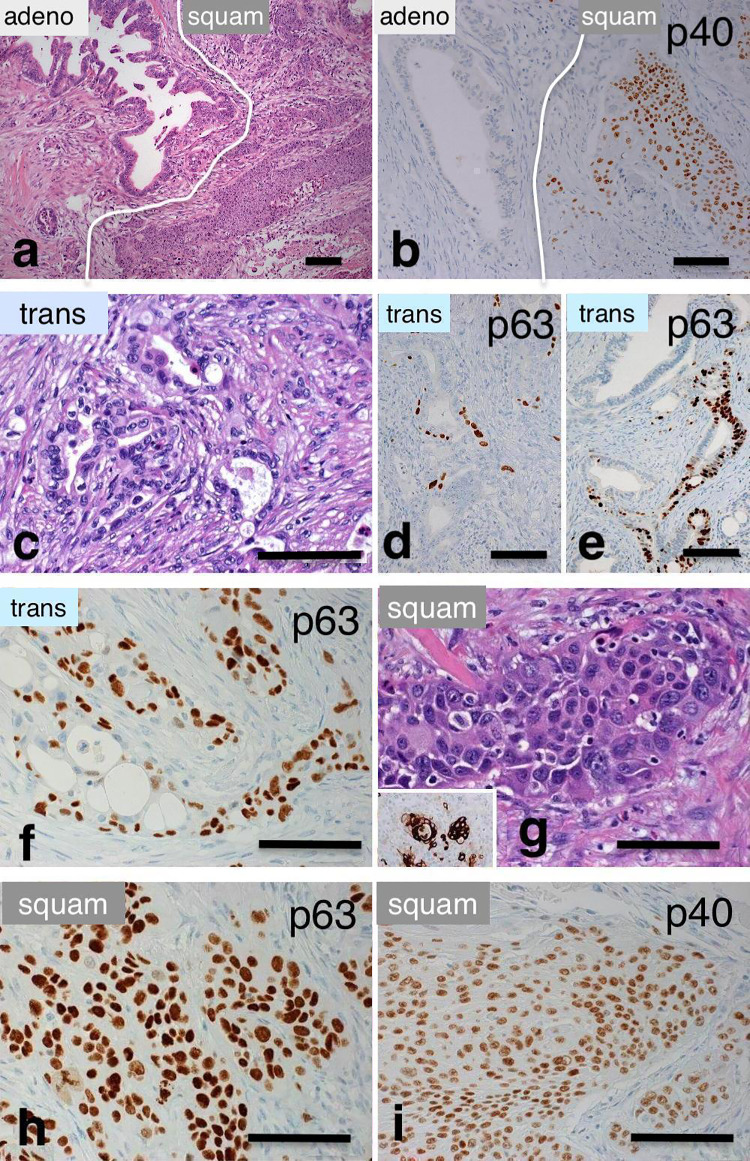
Immunhistological hallmark of the transitional zone in regard to p63- and p40-staining; **a**–**b** typical patchwork pattern of adenosquamous carcinoma in HE-staining, illustrated by p40-negative staining in the glandular component (**b** left) and p40-positivity in the squamous component (**b** right, see also **h** and **i**); **c**–**f** transitional zone with increasing expression of p63 in glandular structures (**d**, **e**, **f**) characterized by the appearance of single or groups of p63 + cells; these cells are partly luminally and partly basally located (compare Fig. 3); **g**–**i** squamous component which shows robustly p63- (**h**) and p40 staining (**i**), inset in (**g**) shows k10/13-positivity in the squamous component. *Adeno* adenocarcinoma; *Squam* squamous carcinoma; *trans* transitional zone. Scale bar 100 µm

ASCAP typically demonstrated a patchwork pattern of adenocarcinoma and squamous carcinoma components of variable grades and proportions, haphazardly arranged in a mosaic pattern (Figs. 1a–c, 2a, b). The entire spectrum of glandular growth and cellular patterns seen in conventional PDACs were observed also in this series of ASCAPs. The squamous components included the features known to occur in conventional squamous carcinoma in other sites. Notably, transitional zones between the glandular and squamous components were identified in most cases (see below).

### p63 and K5/14 positive cells are the likely source of the squamous carcinoma components of ASCAP

**Fig. 3:**
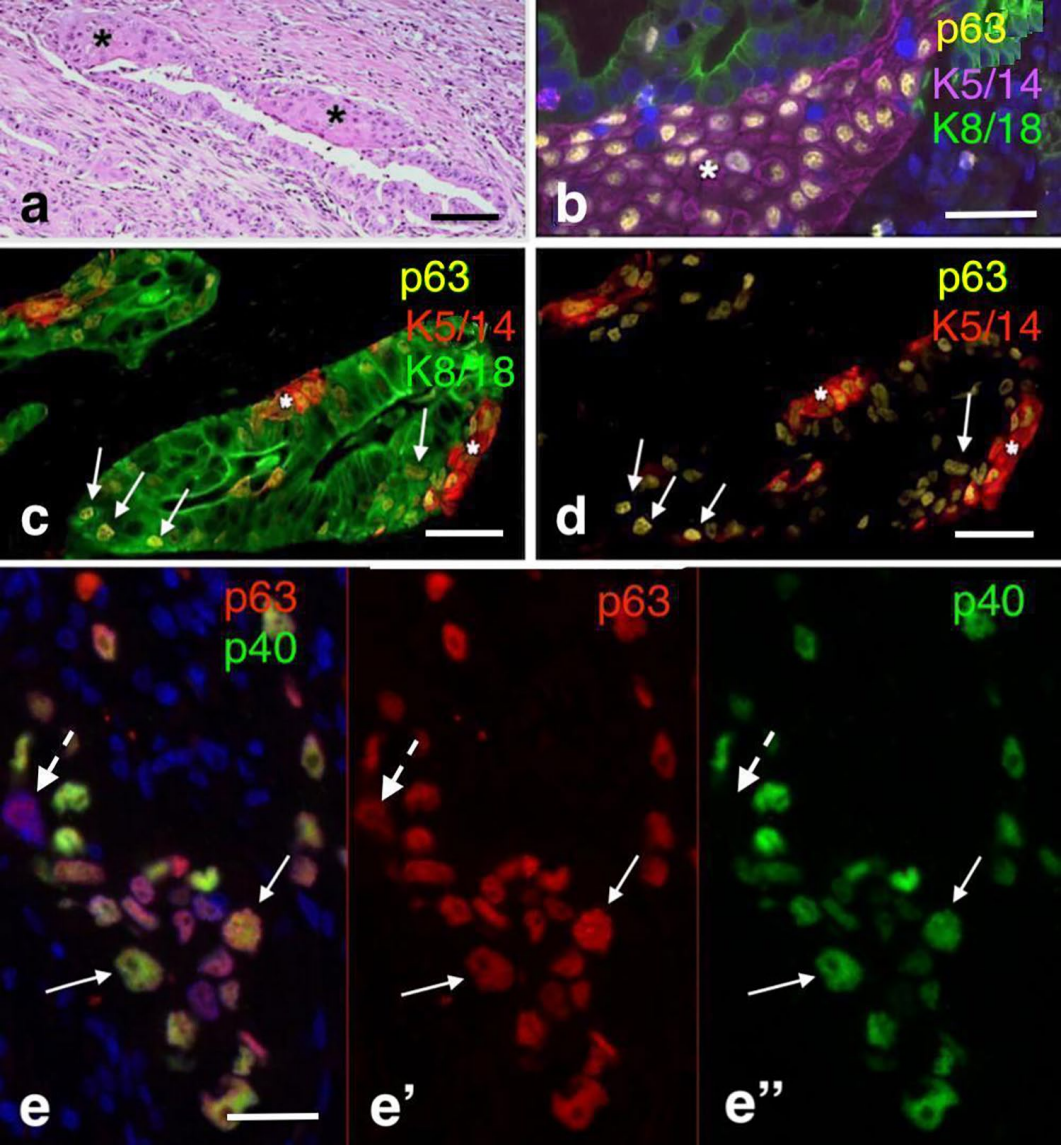
Triple immunostainings demonstrate the process of squamous differentiation in tumour glands; **a**–**b** a tumour gland showing focal squamous differentiation (asterisks) characterized by coexpression of p63 and K5/14; **c**–**d** these pictures demonstrate earlier developments with the expression of p63-positivity in K8/18 + glands (arrows) and basally located p63 + K5/14 + cells (asterisks in **c**–**d**); **e**–**e**’’ Notice that most p63 + cells robustly co-express p40 (arrows). Occasional cells express p63 but lack p40 (broken arrows). Scale bar 100 µm

The immunohistological features of 25 ASCAP and 20 PDAC cases are summarized in Table 3. Strong nuclear p53-immunostaining was observed in 95.8% (23/24) of ASCAP cases as opposed to 60% (12/20) of PDAC-cases. No MUC2 immunostaining was found in ASCAP- and PDAC-cases included in this study. Three different epithelial phenotypic patterns were observed in most tumours of our series of ASCAP cases (Figs. 1, 2, 3 and Supplementary Figs. 2, 3): (1) Areas with pure adenocarcinoma resembling pancreatobiliary-type adenocarcinomas of PDAC was characterized by the expression of K8/18, K7, MUC5AC, MUC1, and CEA. (2) Areas with squamous carcinoma which are positive for p63, p40, K5/14, and variably positive for K10/13. The squamous components overexpressed EGFR in 94.7% of ASCAP cases compared to 20% in PDAC. K8/18 and MUC5AC were not expressed or expressed at low levels in well differentiated squamous carcinoma but were usually expressed, albeit at lower intensity, in poorly differentiated squamous carcinoma; In contrast to the K8/18 staining, decreased positive K7-staining was usually observed in the squamous component. (3) Areas of transitional zones were detected in 16 of the 25 ASCAP cases which were preferentionally localized at the interface between the adenocarcinomatous and the squamous carcinoma areas. These transitional zones are characterized by K8/18 + glandular components that coexpress p63 and, furthermore, show p63+ , K5/14+ cells in basal position (Figs. 2c–f, 3c–e and Supplementary Fig. 1b–d). However, similar phenomena were also found in adenocarcinomatous areas which histologically did not disclose any squamous differentiation. The proliferation of p63+ /K5/14+ cells leads to the formation of squamous structures (Figs. 1, 2, 3 and Supplementary Figs. 1, 2). Importantly, most of the p63 + cells also stain for p40 (Figs. 2h–i, 3e–e”). Taken together, we hypothesize that the p63 + K5/14 + cells are the source of the squamous carcinoma components.

**Table 3:** Immunohistochemical profiling of 20 patients with pancreatic ductal adenocarcinoma (PDAC) and 25 patients with adenosquamous carcinoma of the pancreas (ASCAP)

Marker^a^	p63	p40	K5/14	K10/13	EGFR	K8/18	MUC1	MUC5AC	CEA	p53^b^
PDAC	4/20 (20%)	4/20 (20%)	6/20 (30%)	1/20 (5%)	4/20 (20%)	20/20 (100%)	20/20 (100%)	20/20 (100%)	20/20 (100%)	12/20 (60%)
ASCAP	25/25 (100%)	25/25 (100%)	25/25 (100%)	16/25 (64%)	18/19 (94.7%)	25/25 (100%)	25/25 (100%)	17/20 (85)	25/25 (100%)	23/24 (95.8%)

^a^Cut-off value 5%

^b^Diffuse positive staining in both glandular and squamous component

Pancreatic Intraepithelial Neoplasia 3 (PanIN 3) was observed in 52% of the ASCAP cases. In one of these, a focal positivity for p63+ and K5/14+ of a PanIN lesion was found in pancreatic tissue not involved by invasive cancer (Supplementary Fig. 3).

### PDAC and associated PanIN 3 lesions contain foci of p63+ and k5/14+ tumour cells similar to the transitional zone in ASCAP

Twenty-five percent of conventional PDACs without any histological evidence of squamous differentiation contain foci of p63+ and K5/14+ cells (Supplementary Fig. 5) with a median of 15% of p63-immunopositive tumour cells (range 10–25%). Associated PanIN 3 lesions were found in 10 cases (50%) of PDAC. In one of these lesions p63+ , K5/14+ cells were found which even expressed the squamous keratins K10/13 and EGFR in a histologically classical PanIN 3 lesion.

## Discussion

Here, we aim to get further insight into the histogenesis of ASCAP by analysing the cellular composition and differentiation of these lesions using immunohistochemistry and triple immunofluorescence stainings. The adenocarcinomatous component shows a pronounced morphological and immunophenotypic similarity to classical PDAC with positivity for K8/18, MUC1, and MUC5AC. Notably, the squamous lineage of these lesions is identical to squamous carcinomas in other anatomical locations and its hallmark is the robust positive staining for p63 (Uramoto et al. 2010), p40, and K5/14 in all cases, with coexpression of the EGFR in 94.7%, and variable staining for the squamous keratins K10/13.

**Fig. 4:**
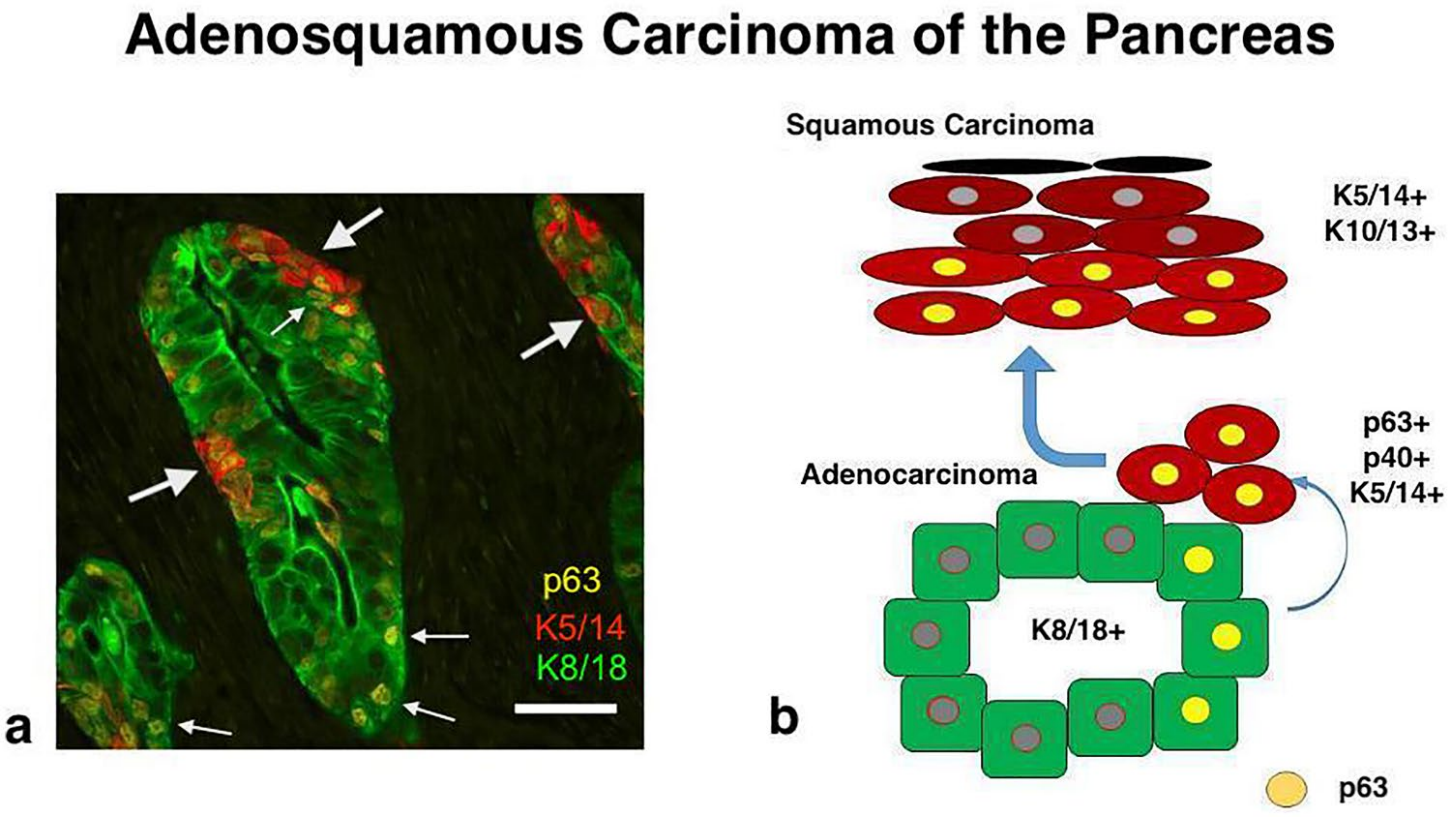
Hypothetical model of adenosquamous carcinoma of the pancreas a: triple immunofluorescence demonstrating the expression of p63 in the k8/18 + glandular cells (small arrows) and the basally located p63 + K5/14 + cells (large arrows) (this figure contains the same epithelial structure as in Fig. 3c); **b** hypothetical cellular model explaining the development of the glandular and squamous components in these tumours. Scale bar 100 µm

Based on the data of our study, we present new evidence in support of a transdifferentiation of adenocarcinoma to squamous carcinoma in ASCAP consistent with the squamous metaplasia theory (Cihak et al. 1972) (Fig. 4). This hypothesis is supported by the presence of clearly recognizable transitional zones between the K8/18 + MUC5AC + adenocarcinomatous and the p63 + K5/14+ squamous components. The transitional zone is characterized by the early appearance of nuclear p63 expression in K8/18+ glandular cells and the occurrence of p63 + K5/14+ basally located cells. These cells subsequently proliferate and differentiate forming the squamous components of ASCAP. Notably, in all cases most of the p63 + cells stained robustly for p40 (Fig. 3) and in 64% also for the squamous keratins K10/13. Thus, it seems as if the K8/18 + glandular cells of ASCAP transform to p63 + /p40 + /K5/14 + cells which, therefore, constitute the squamous metaplastic epithelial proliferations in these tumours. The findings indicate that p63 and p40 are key players in this metaplastic process. This is in line with reports showing that deltaNp63, i.e. p40, is the most widely expressed isoform in squamous carcinoma, compatible with a role for this protein in promoting the growth of neoplastic cell in these tissues (Nylander et al. 2002; Reis-Filho et al. 2002).

Our interpretation finds its analogy in the embryogenetic development of several epithelia (Daniely et al. 2004; Di Como et al. 2002; Koster et al. 2004; Koster and Roop 2004b; Signoretti et al. 2000). Early embryonic mouse ectoderm shows transformation of single layered K8/18+ cells to multi-layered squamous epithelium with p63-positivity in basal keratinocytes (Koster and Roop 2004a). In this context p63 appears to initiate epithelial stratification and to maintain proliferative potential of basal keratinocytes in mature squamous epithelium (Senoo et al. 2007). Similarly, the tracheobronchial epithelium in mice consists of a layer of two to three cells. During development, stem cells first differentiate into p63-negative ciliated and secretory cells and later, at birth, they begin to differentiate into p63 + K14 + basal cells (Daniely et al. 2004).

Additional evidence in favour of the squamous metaplasia theory for ASCAP comes from our finding that one fourth of unselected cases of the PDACs without histologically recognizable squamous differentiation contained small areas with p63+ , and K5/14 + cells and even p40+ and K10/13+ cell differentiations. Expression of squamous type keratins in PDAC and their possible relation to squamous metaplasia have already been discussed as early as 1989 (Moll et al. 1989; Schussler et al. 1992). Collectively, we, therefore, suggest that these findings are indicative of an abortive squamous metaplasia in otherwise classical PDACs and we suggest that these findings might express the metaplastic potential of the neoplastic ductal epithelium.

Furthermore, 54% of our ASCAP-cases were associated with PanIN 3 changes. As most invasive PDACs are thought to develop through the adenoma-carcinoma pathway and, based on the assumption that the squamous component originates from the adenocarcinoma, it is reasonable to postulate a developmental sequence from PanIN via PDAC to ASCAP. In line with this argument are case reports that have described the association of ASCAP with intraductal papillary mucinous neoplasia and suggested a model of adenoma-carcinoma progression for these tumours (Martinez de Juan et al. 2017; Matsuzaka et al. 2016).

Finally, indirect evidence in favour of our model comes from previous studies showing that several glandular epithelia which contain p63 + K5/14 + basal cells do not express p63 in the differentiated glandular compartment (Boecker et al. 2019; 2014; Daniely et al. 2004; Di Como et al. 2002; Nylander et al. 2002; Signoretti et al. 2000). Furthermore, in contrast to the findings in this ASCAP study, low-grade adenosquamous carcinoma/syringomatous tumours of the breast, which contain p63 + and K5/14 + cells, first downregulate p63 and subsequently generate the K8/18 + glandularly differentiated cells via p63-negative K5/14 + intermediary cells indicating a different histogenetic pathway in these lesions compared to ASCAP tumours (Boecker et al. 2019; 2014).

In conclusion, based on the findings of the cellular organization of ASCAP in this study, we subscribe to the theory that these tumours occur as a result of malignant squamous metaplastic change of an adenocarcinoma. Our findings suggest that the squamous carcinoma component originates from a pre-existing K8/18 + PDAC through transdifferentiation of glandular cells to p63+ , p40+ , and K5/14 + squamous carcinoma cells. Additional studies will be needed to fully understand the molecular mechanisms behind these interesting cellular differentiation processes.

## Electronic supplementary material

Below is the link to the electronic supplementary material.

Immunohistological features of transitory areas in **a**–**d** contrasting with a differentiated squamous component in **e**–**h**; **a**–**d** transitory area with adenocarcinomatous component containing complex glandular structures in HE-stain (**a**) and single or small clusters of p63+ and K5/14+ cells (**b**–**d**); **e**–**h** squamous differentiation found in an otherwise typical glandular structures in HE-stain (**e**) with positivity for p63 (**f**), K5/14 (**g**) and even the squamous keratins K10/13 (**h**) indicating the squamous differentiation. Scale bar 100 µm (JPG 280 kb)

Triple immunostainings demonstrate the positive p63- and K5/14-staining of the squamous differentiation in two tumour glands; **a**–**b** and **c**–**d**: tumour glands showing squamous differentiations (asterisks) characterized by coexpression of p63 and K5/14. Scale bar 100 µm (JPG 177 kb)

PanIN 3 associated with adenosquamous carcinoma. This picture demonstrates PanIN 3 with micropapillary growth (**a**) with a cluster of cells showing p63 (**b**) and K5/14-expression (**c**). Notice the high Ki67- proliferation index of the entire epithelium (inset in **a**). Scale bar 100 µm (JPG 299 kb)

Pancreatic ductal adenocarcinoma, pancreatobiliary-type consisting of simple glands with pale-staining tumour cells (**a**) and more complex glands (**b**); This picture demonstrates focal expression of p63 (**c**–**d**) and K5/14 (**e**–**f**) which was found in less than 10% of the tumour cells. The tumour cells stain strongly for K8/18 (g). Scale bar 100 µm (JPG 280 kb)

## Abbreviations

ASCAP: Adenosquamous carcinoma of the pancreas
PDAC: Pancreatic ductal adenocarcinoma
K: Keratin
CEA: Carcino-embryonal antigen
