# Supplementary figures and images for: Cellular organization and histogenesis of adenosquamous carcinoma of the pancreas: evidence supporting the squamous metaplasia concept
Source: Histochem Cell Biol. 2020 Mar 13;154(1):97–105. doi: 10.1007/s00418-020-01864-y (PMC7343762; doi:10.1007/s00418-020-01864-y)

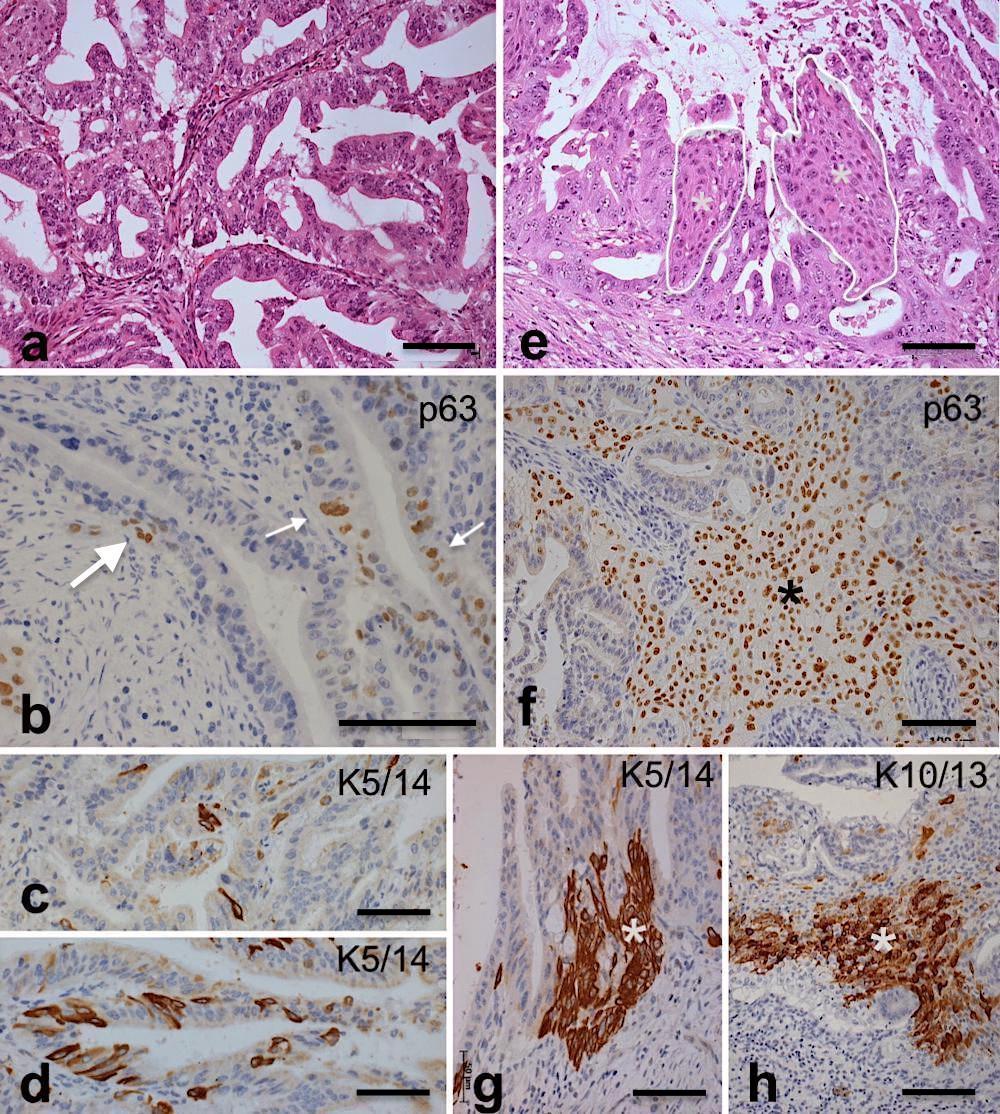

Supplement: Supplementary file 1 — Immunohistological features of transitory areas in a–d contrasting with a differentiated squamous component in e–h; a–d transitory area with adenocarcinomatous component containing complex glandular structures in HE-stain (a) and single or small clusters of p63+ and K5/14+ cells (b–d); e–h squamous differentiation found in an otherwise typical glandular structures in HE-stain (e) with positivity for p63 (f), K5/14 (g) and even the squamous keratins K10/13 (h) indicating the squamous differentiation. Scale bar 100 µm (JPG 280 kb) [file 418_2020_1864_MOESM1_ESM.jpg]

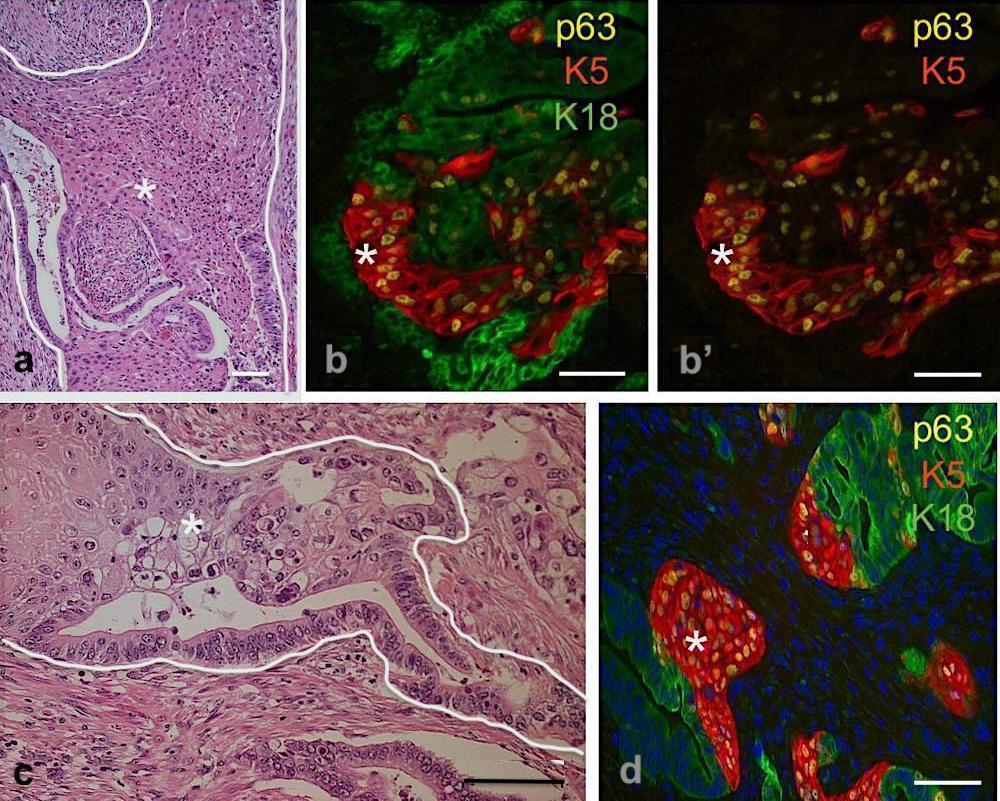

Supplement: Supplementary file 2 — Triple immunostainings demonstrate the positive p63- and K5/14-staining of the squamous differentiation in two tumour glands; a–b and c–d: tumour glands showing squamous differentiations (asterisks) characterized by coexpression of p63 and K5/14. Scale bar 100 µm (JPG 177 kb) [file 418_2020_1864_MOESM2_ESM.jpg]

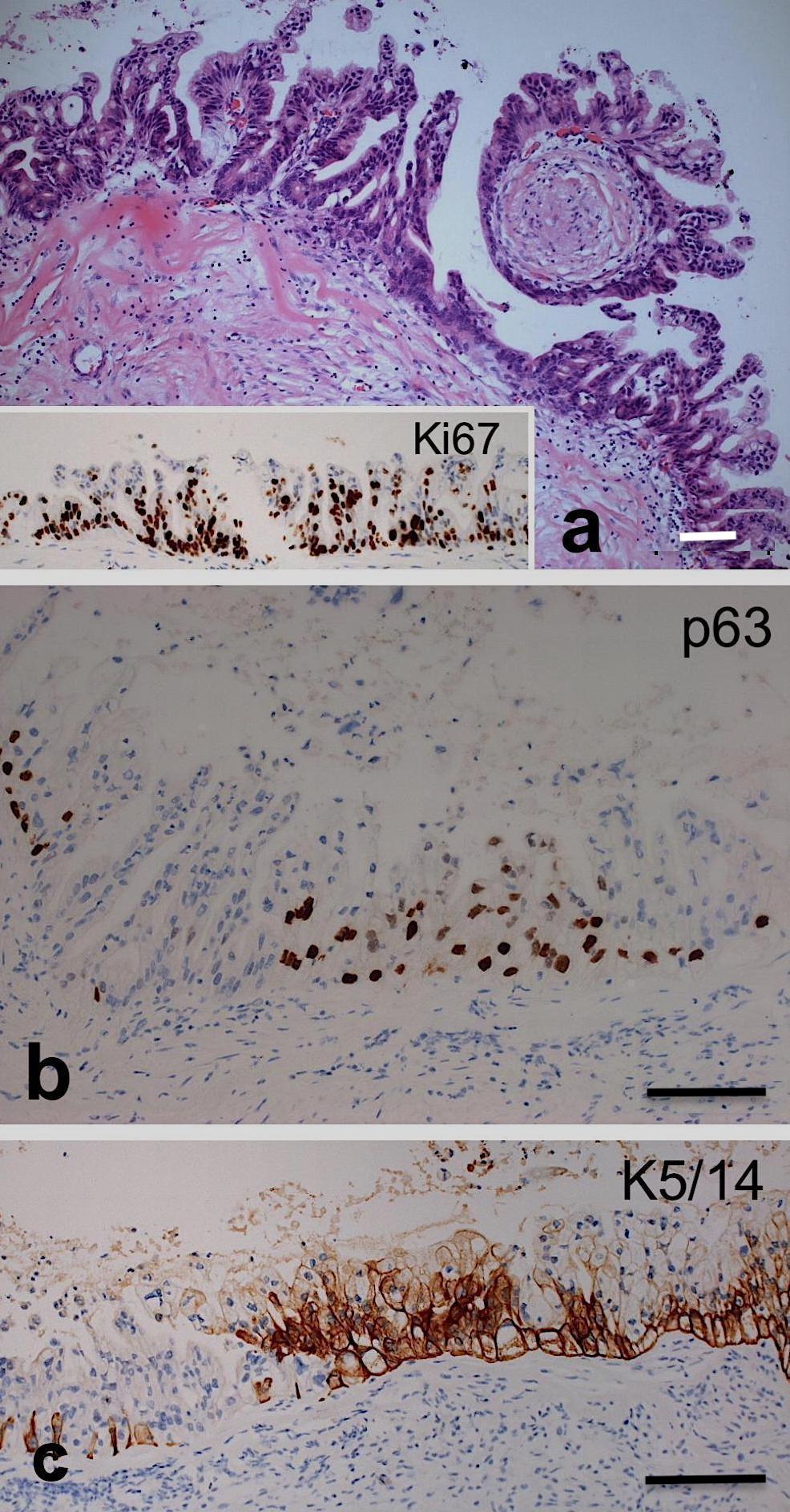

Supplement: Supplementary file 3 — PanIN 3 associated with adenosquamous carcinoma. This picture demonstrates PanIN 3 with micropapillary growth (a) with a cluster of cells showing p63 (b) and K5/14-expression (c). Notice the high Ki67- proliferation index of the entire epithelium (inset in a). Scale bar 100 µm (JPG 299 kb) [file 418_2020_1864_MOESM3_ESM.jpg]

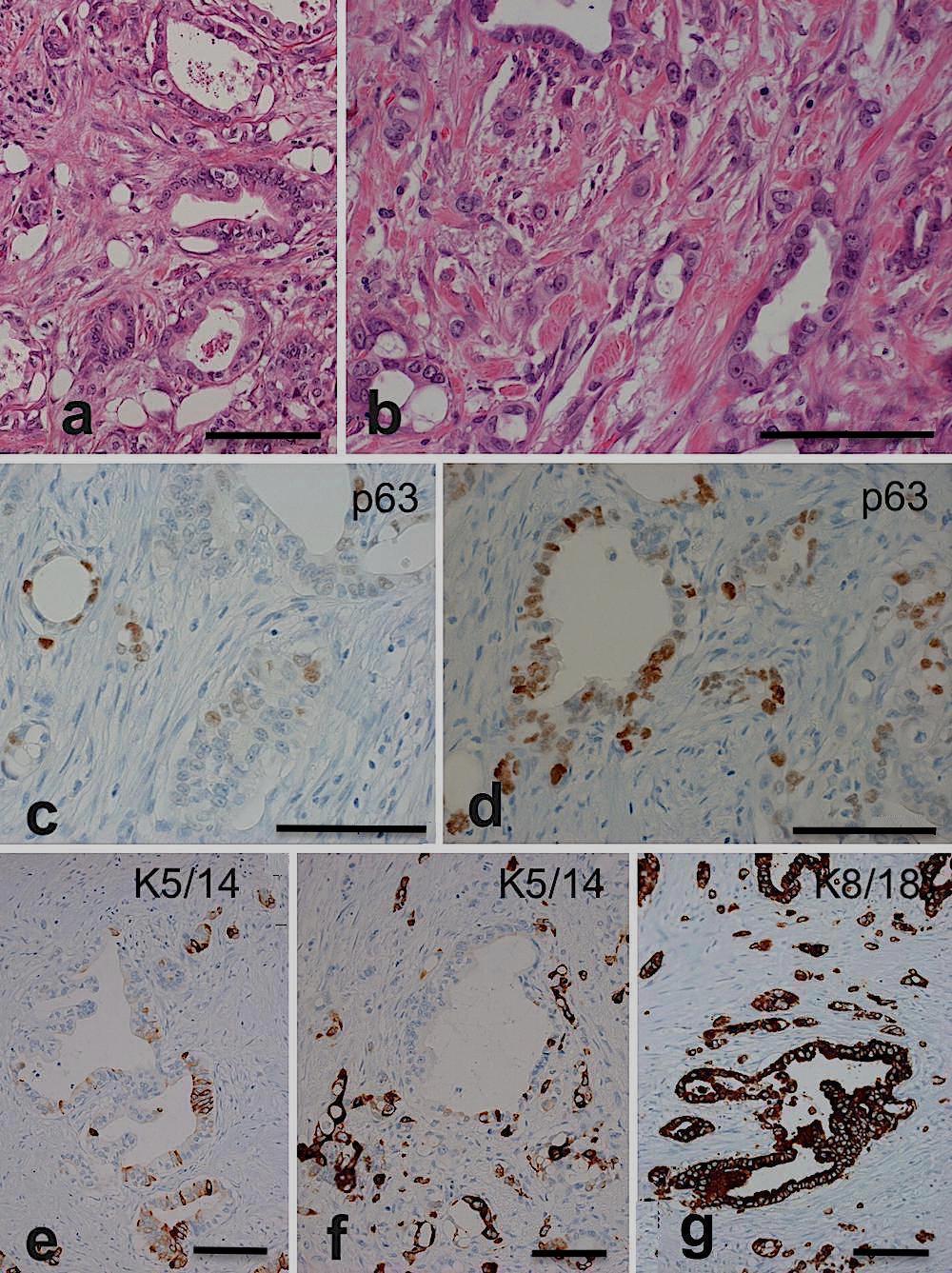

Supplement: Supplementary file 4 — Pancreatic ductal adenocarcinoma, pancreatobiliary-type consisting of simple glands with pale-staining tumour cells (a) and more complex glands (b); This picture demonstrates focal expression of p63 (c–d) and K5/14 (e–f) which was found in less than 10% of the tumour cells. The tumour cells stain strongly for K8/18 (g). Scale bar 100 µm (JPG 280 kb) [file 418_2020_1864_MOESM4_ESM.jpg]
